# Effect of Systemic Prednisone Treatment on Changes of Inflammation Markers in Chronic Rhinosinusitis

**DOI:** 10.3390/diagnostics13071354

**Published:** 2023-04-05

**Authors:** Malgorzata Wierzchowska, Paulina Kalińczak-Górna, Magdalena Zwolińska, Joanna Ligmanowska, Justyna Durślewicz, Aleksander Zwierz, Bartosz Malinowski, Michał Wiciński, Paweł Burduk

**Affiliations:** 1 Department of Otolaryngology, Phoniatrics and Audiology, Collegium Medicum, Nicolaus Copernicus University, 85-067 Bydgoszcz, Poland; 2 Department of Otolaryngology, The Provincial Polyclinical Hospital in Toruń, 87-100 Toruń, Poland; 3 Department of Clinical Pathomorphology, Collegium Medicum, Nicolaus Copernicus University, 85-067 Bydgoszcz, Poland; 4 Department of Pathophysiology, Collegium Medicum, Nicolaus Copernicus University, 85-067 Bydgoszcz, Poland; 5 Department of Pharmacology and Therapy, Collegium Medicum, Nicolaus Copernicus University, 85-067 Bydgoszcz, Poland

**Keywords:** rhinosinusitis, inflammation, FESS, prednisone, steroid

## Abstract

(1) Objective: We aimed to evaluate the effect of treatment with prednisone on nasal and systemic periostin and eotaxin expression, IgE in plasma and eosinophils in tissue. (2) Methods: We compared the values of nasal and systemic periostin, eotaxin, IgE and eosinophils in tissue in patients treated with only nasal steroids before FESS, group 1, with those treated with an oral steroid–prednisone, group 2. (3) Results: A statistically significant decrease in the level of periostin, eotaxin and IgE in plasma was achieved in patients treated with prednisone one week before and after surgery (in sequence: *p* < 0.0476, *p* < 0.0006, *p* < 0.0031). In patients treated with steroids, we also observed a lower level of periostin in the epithelium (*p* < 0.044), eotaxin in the stroma (*p* limit value < 0.075) and eosinophils (*p* < 0.031) in the tissues collected during the operation. (4) Conclusions: Systemic steroid treatment with prednisone distinctly decreases periostin, eotaxin and IgE expression in plasma. We also observed a lower level of periostin in the epithelium, eotaxin in the stroma and eosinophils in the tissues. We need more attempts to find inflammatory markers associated with chronic rhinosinusitis. Identifying drugs that decrease inflammatory parameters would allow for more targeted therapy.

## 1. Introduction

Chronic rhinosinusitis (CRS) is a common, complex disease with an undetermined etiology. Due to its chronic and recurrent nature, it is a serious health problem. The pathogenesis of CRS involves a number of interdependent factors. The complex etiology causes difficulties in choosing the right treatment and leads to recurrence of the disease. It is characterized by at least two symptoms, one of which should be either nasal blockage/obstruction/congestion or nasal discharge (anterior/posterior nasal drip). Other symptoms include pain or a feeling of pressure in the face, and a reduction or loss of smell. The European Position Paper on Rhinosinusitis (EPOS) 2020 defined chronic rhinosinusitis as an inflammation process of the nose and paranasal sinuses mucosa characterized by chronic symptoms lasting more than 12 weeks. Among the symptoms visible in nasal endoscopy, we can evaluate the presence of nasal polyps and/or mucopurulent discharge and/or swelling of the mucosa (especially in the middle nasal meatus) [1].

Although the cause of CRS remains unclear, mucosal inflammation and infiltrating inflammatory cell profiles (eosinophils or neutrophils, differentiated T cell patterns) and tissue remodeling might be helpful to identify disease entities [2]. Tissue eosinophilia is a well-known predictor of a poor prognosis associated with a high recurrence of the disease, persistent postoperative symptoms and unsatisfactory healing [3]. The changes caused by tissue remodeling in CRS such as squamous metaplasia, basement membrane (BM) thickening, stromal edema and fibrosis, goblet cell hyperplasia and subepithelial gland hyperplasia are the main factors affecting proper healing [4]. The relationship between histologic changes and inflammatory markers in CRS remains unclear, so we need more research to find different inflammatory markers associated with chronic rhinosinusitis. By defining such diagnostic exponents, we can provide more information about future treatment.

Periostin is a new marker with special expression in chronic rhinosinusitis. Periostin (POSTN) is a protein secreted mainly by collagen-rich connective tissues, such as periosteum, periodontal ligaments, tendons, heart valves and skin. It is an extracellular matrix protein that interacts with the ECM (extracellular matrix) and plays a role in the pathologic remodeling changes, particularly in eosinophilic inflammation [1]. Periostin interacts with several integrin molecules (αvβ1, αvβ3 and αvβ5) on cell surfaces, providing signals for tissue development and remodeling. In the literature, it has been found that periostin is a product of IL-4 or IL-13, and activates Th2-type cytokines in immune responses [4]. It was proven that periostin accelerates eosinophil recruitment and activation and is involved in allergic inflammation and eosinophil-mediated inflammation. On the other hand, RANTES and eotaxin-2 (the CC family of chemokines) are particularly significant to eosinophils’ activation and infiltration during NP formation [5,6]. However, the mechanism by which eosinophils are selectively recruited in nasal polyps remains to be clarified, and whether periostin might influence the tissue eosinophilia of ENP by modulating eotaxin-2 and RANTES secretion is not clear and warrants further investigation. Periostin is present in the thickened basement membrane and in the serum of asthmatic patients, especially those with eosinophilic airway inflammation and atopy. Periostin induces hypersecretion of type 2 inflammatory factors and mucus production, and is associated with asthma, allergic rhinitis and CRS [7]. It is also present in the sinonasal mucosa, nasal polyps and serum. It is highly expressed in eosinophilic CRSwNP (CRS with nasal polyps) and correlates with the severity of inflammatory changes [7]. Serum periostin levels in asthma patients decrease in response to therapy with systemic or inhaled corticosteroids. The measurement of periostin serum levels in clinical practice could be a good factor for monitoring treatment effects. The use of isoforms specific for chronic rhinosinusitis, as well as the standardization of assay methods, may enable an objective determination of the clinical usefulness of periostin as a CRS marker [7,8,9].

On the other hand, eotaxin is a member of the C-C family of chemokinesis, and cytokine is produced by the epithelium and can induce the chemotaxis of eosinophils, basophils and TH2 lymphocytes. Eotaxin is a more specific activator of the selective migration of inflammatory cells in relation to other cytokines. Numerous reports indicate that eotaxin CCR3 is important in the accumulation of eosinophils in tissues and, consequently, in the intensification of the inflammatory process. Due to its multiform effect on eosinophils, eotaxin is considered to be one of the main mediators of the formation of eosinophilic infiltrates and the development of inflammation. Eotaxin, interacting with interleukin-5, increases the number of mature eosinophils in the blood, which contributes to the formation of peripheral and tissue eosinophilia. It plays an indirect role in airway remodeling through the recruitment of eosinophils and mast cells, which have profibrogenic and proangiogenic activities, into the site of inflammation [9]. Its level has been shown to be significantly elevated in patients with more than 55 tissue eosinophils per HPF (high-power field, in microscopy) and may be important as a marker for disease recurrence [10].

The role of tissue eosinophilia as a marker predicting CRS recurrence has been proven in many publications. Greater tissue eosinophilia had a greater risk of recurrence and unsatisfactory treatment outcomes were observed after sinus surgery, such as fast polyp growth, crusting and mucopurulent discharge. However, neither a clear definition of nor diagnostic criteria for eosinophilic CRS have been established. Previous investigations consider that more than 55 eosinophils per high-power field was suggested as the cutoff eosinophil count for eosinophilic CRS [3].

Blood eosinophil counts greater than 0.24 × 10^9^/L suggest an eCRS (eosinophilis CRS) with tissue eosinophilia greater than 10 eosinophils per high-power field [11]. In the case of blood eosinophil counts above 0.45 × 10/L, there is a need for long-term systemic treatment after endoscopic sinus surgery [12]. Additionally, blood eosinophil is associated with a need for long-term systemic therapy following endoscopic sinus surgery [13].

Treatment with local glucocorticoids has proven anti-inflammatory effects. Topical glucocorticosteroids, due to their strong, multidirectional anti-inflammatory effect, play a key role in the treatment of chronic rhinosinusitis. They reduce the release of eosinophils by the bone marrow and inhibit their survival time. They have strong vasoconstrictive properties, which reduces swelling and exudate. There is strong evidence for the use of nasal steroids for chronic rhinosinusitis, especially for the treatment of nasal polyps, which is the standard first-line treatment. There are reports suggesting that glucocorticosteroids inhibit periostin expression [7].

The International consensus statement on allergy and rhinology: rhino sinusitis 2021 (2021 ICAR-RS) statement on rhinosinusitis states that oral corticosteroids are an option in CRS treatment, but still cites a major lack of high-level evidence [14]. Despite the indications for the use of oral corticosteroids, there are still unknowns about their potential harmfulness. The conducted studies indicate no differences between short-term, oral and inhaled corticosteroids in terms of the biochemical suppression of the hypothalamic–pituitary–adrenal axis.

There are no clear guidelines for which patients should be given oral corticosteroids. In case of insufficient disease control, oral corticosteroid courses are usually prescribed. However, the development of optimal doses and duration of corticosteroid therapy still requires further research.

We aimed to evaluate the real effect of treatment with prednisone on nasal tissue and systemic periostin and eotaxin, IgE level in plasma and eosinophil count in tissues.

## 2. Materials and Methods

### 2.1. Material Collection and Preparation

We compared the values of nasal (in epithelium and stroma) and systemic (in plasma) periostin and eotaxin levels, IgE in plasma and eosinophils in tissues in patients treated with only nasal steroids before FESS (functional endoscopic sinus surgery), group 1 (*n* = 15), with those treated with the oral steroid prednisone, group 2 (*n* = 15). Patients in group 2 took 40 mg of prednisone once daily in the week before surgery, and the doses were gradually decreased during the week after the FESS procedure according to the scheme 30 mg for 3 days, 20 mg for 2 days, 10 mg for 1 day and 5 mg on the last day.

Blood samples for the assessment of marker’s levels were collected one week before the operation (which we called point A), after admission to the hospital (point B) and also 1 month after the procedure (point 3). Markers such as periostin, eotaxin and IgE were measured by means of an immunoassay (ELISA) in preserved serum samples and nasal secretions. Serum tubes were collected and centrifuged for 20 min. The plasma was then pipetted into Eppendorf tubes and stored at −80 °C.

Mucosal tissues from patients with CRS were obtained from the nasal polyps or inflammatory tissue of the ethmoid cavity during surgery and underwent standard hematoxylin and eosin (H&E) staining.

### 2.2. H&E and Immunohistochemical Staining

Formalin-fixed, paraffin-embedded (FFPE) tissue blocks were cut into 5 µm and 4 µm thick sections for hematoxylin and eosin (H&E) and immunohistochemical staining, respectively. For this step, a rotary microtome was used (Accu-Cut^®^ SRM™ 200; Sakura, Torrance, CA, USA). The prepared sections were then transferred onto slides and left for 1 h on a heating plate set at 60 °C.

**Figure 1:**
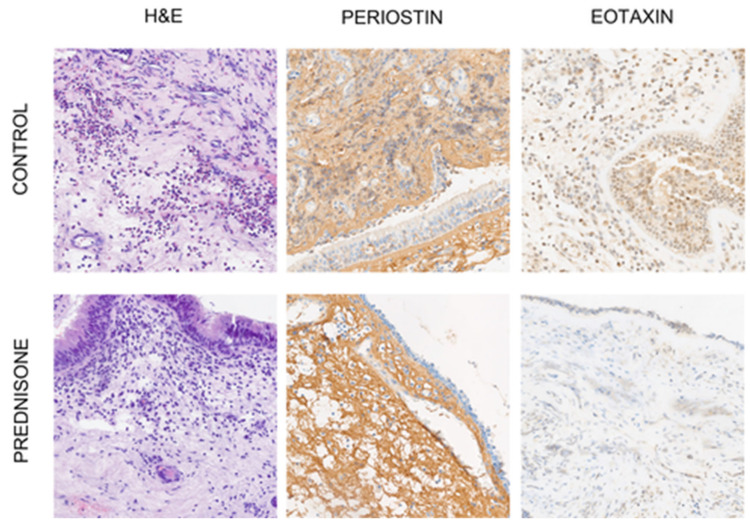
Hematoxylin and eosin staining, periostin and eotaxin expression. Control group compared to group treated with prednisone.

H&E staining was used to evaluate the cellular content, especially eosinophils. The immunohistochemical staining of periostin and eotaxin was performed automatically in a BenchMark Ultra Platform (Ventana Medical System, Indianapolis, IN, USA). The antibody was previously prepared manually and added in the appropriate step of staining. The reaction was detected using a visualization system (Ultra View DAB Detection Kit; Ventana Medical System, Oro Valley, AZ, USA, Figure 1). Finally, the slides were dehydrated in an alcohol gradient, cleared in xylenes and mounted with Shandon Consul-Mount (Thermo Scientific, Waltham, MA, USA).

### 2.3. Expression Analysis

The immunohistochemical evaluation of protein expression was performed in a blinded fashion by two independent pathologists using a light ECLIPSE E400 microscope (Nikon Instruments Europe, Amsterdam, The Netherlands).

The evaluation of eosinophils was carried out at 40× original objective magnification in one place with representative expression of the so-called hot-spot. The number of eosinophils in the representative tissue section was classified as follows: (1) up to 10 cells/area; (2) 11–50 cells/area; (3) 51–100 cells/area; (4) equal or more than 100 cells/area.

The expression of periostin and eotaxin was analyzed at 40× original objective magnification and according to the modified index Remmele–Stegner (IRS) scale [8], in which the percentage of positively stained cells/areas is multiplied by the intensity of staining. The scores for positive immunoreactivity were categorized as follows: (0) less than 5% of stained cells/area; (1) 6–25% of stained cells/area; (2) 26–50% of stained cells/area; (3) 51–75% of stained cell/area and (4) equal to or more than 75% of stained cells/area, whereas the staining intensity was evaluated using the following criteria: (0) negative; (1) low staining; (2) moderate staining and (3) strong staining. The final staining score range from 0 to 12.

The study protocol was approved by the local ethics committee (approval number: KB 138/2019). This examination was conducted in accordance with the Helsinki Declaration.

The exclusion criteria to the study were patients with other systemic diseases (metabolic diseases, cardiovascular diseases), neoplasms and previous surgical procedures in the period of 6 months before enrollment in the study. The study groups were comparable in terms of sex and age.

### 2.4. Statistical Analysis

Statistical analysis was carried out using GraphPad Prism version 7.01 (GraphPad Software, La Jolla, CA, USA). The Shapiro–Wilk test was used to verify the normality of the data. The Mann–Whitney test and the Wilcoxon signed rank test were used to compare continuous variables. To assess the correlation between the variables investigated, the Spearman correlation coefficient was used. A *p* value of less than 0.05 was considered statistically significant.

## 3. Results

**Figure 2:**
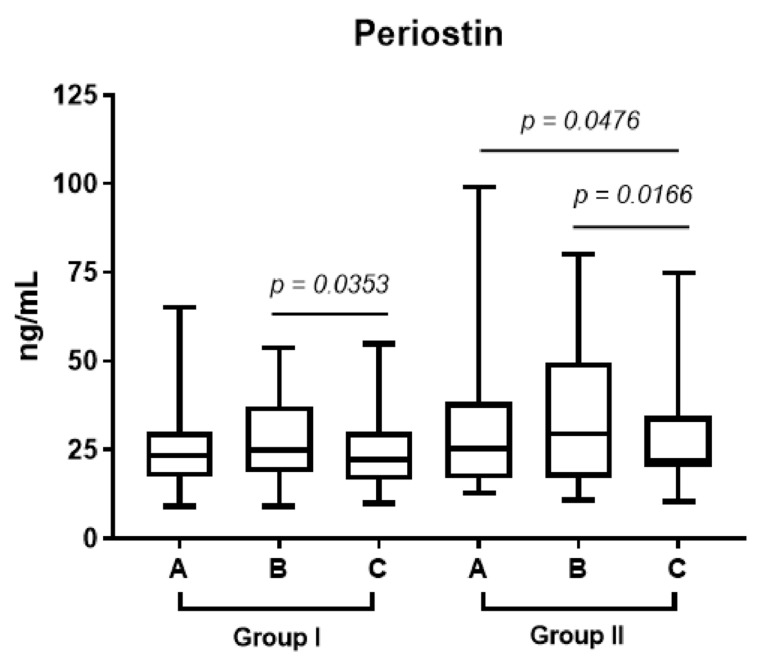
The median level of periostin in plasma one week before operation (point A), after admission to the hospital (point B) and 1 month after FESS (point C) in groups 1 and 2.

In both groups, the level of periostin increased by the end of the week before the surgery (point B). However, in group 2, who received prednisone, the level of periostin in plasma (point C vs. point B in Figure 2; *p* < 0.017) decreased significantly one month after FESS.

**Figure 3:**
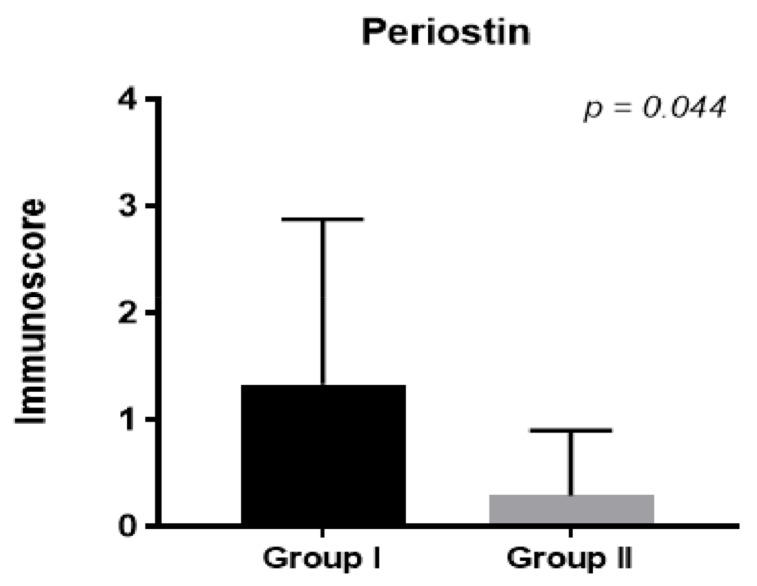
Medium periostin level in epithelium in groups 1 and 2.

In our study, the plasma and epithelium periostin levels in patients receiving prednisone decreased significantly one month after operation compared to the control group (*p* < 0.017; Figure 2; *p* < 0.044, Figure 3).

This was correlated with better healing after surgery, mostly shown by lower scores in the Lund–Kennedy endoscopic scale (Table 1). By performing an endoscopic examination of both nasal cavities separately, we assessed the presence and intensity of edema, polyps and secretions in the nasal meatus using the Lund–Kennedy scale. We obtained a score in the range of 0–12 points. In patients treated with prednisone, we observed less crusting, edema and polyp formation.

Additionally, in patients treated with steroids, the periostin level in the epithelium decreased one month after FESS compared to the control group (Figure 3, *p* < 0.044). We did not observe significant differences in the stroma (*p* < NS).

**Figure 4:**
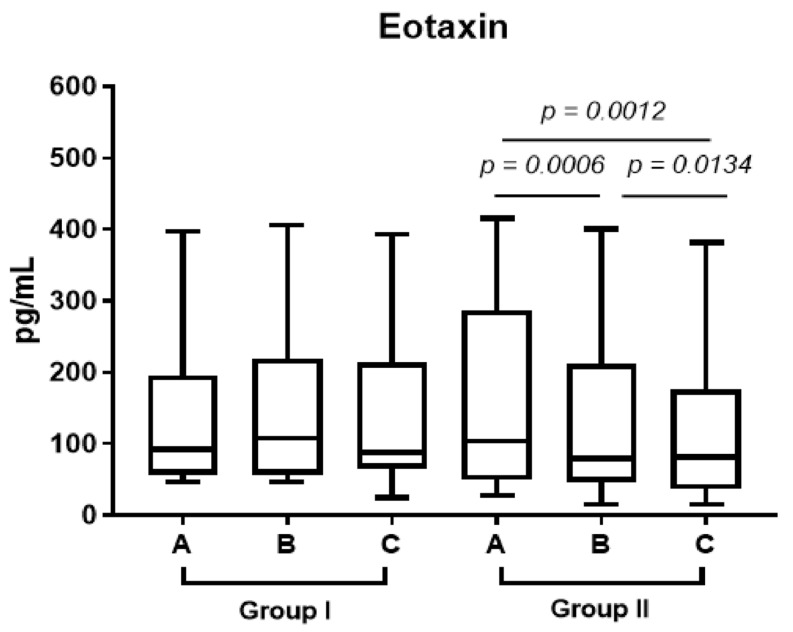
The median level of eotaxin in plasma one week before operation (point A), after admission to the hospital (point B) and 1 month after FESS (point C) in groups 1 and 2.

In group 2, eotaxin decreased in the plasma after one week’s treatment with prednisone and one month after surgery (Figure 4, A vs. B *p* < 0.0006, B vs. C *p* < 0.0134).

In patients treated with steroids, we observed a lower level of eotaxin in the stroma compared to the control group (*p* limit value < 0.075). We did not observe significant differences in the epithelium (*p* < NS).

In group 2, the level of IgE in the plasma (point C vs. point B, *p* < 0.0031) decreased significantly one month after FESS. We did not observe significant differences at the end of the week preceding operation (*p* < NS).

**Figure 5:**
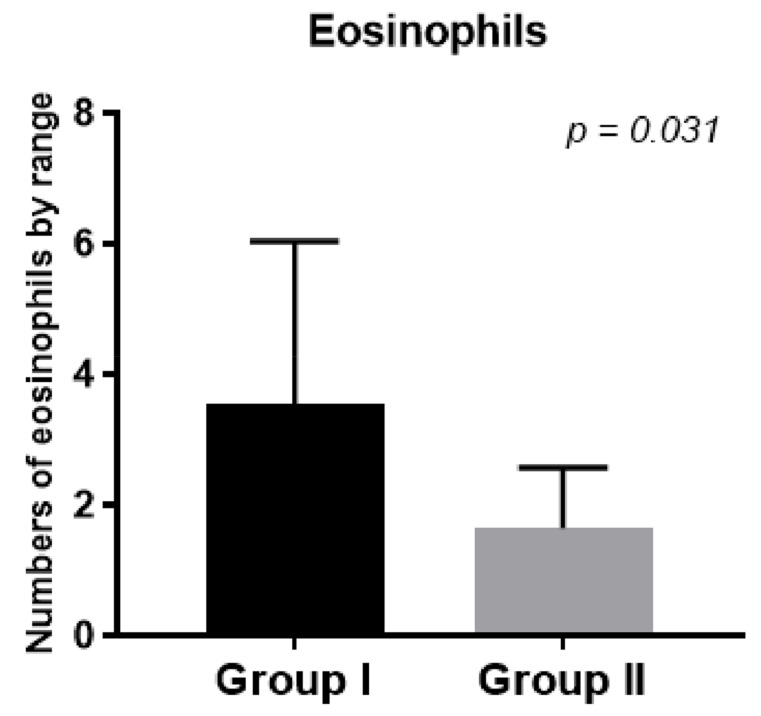
Eosinophil numbers in tissues collected during FESS in groups 1 and 2.

In the second group, eosinophil numbers in the tissues collected during FESS decreased after prednisone treatment compared to the first control group (Figure 5, *p* < 0.031).

We did not observe significant differences between the levels of the tested markers in group 1 at any point in the study, both in the plasma and in the tissues tested (*p* < NS, Table 1).

## 4. Discussion

The pathogenesis of CRS involves a number of interdependent factors, and complex etiology causes difficulties in choosing the right treatment and implies recurrence of the disease. New trials are needed to find inflammatory markers that correlate with the severity of changes in chronic paranasal rhinosinusitis and whose levels are related to the medications used in chronic rhinosinusitis treatment. The 2021 ICAR (International consensus statement on allergy and rhinology) declares that oral corticosteroids are an option, but still cites a major lack of high-level evidence [14]. CRS, especially with nasal polyps or eosinophilia, is difficult to treat. The identification of inflammatory mediators can help in the prognosis and appropriate treatment of CRS.

Normally, corticosteroids have been the mainstay of medical treatment for patients with CRSwNP [15,16]. Corticosteroids can be administered locally within the sinonasal cavity through intra-nasal sprays, sinus rinses or implanted devices, or systemically via oral or parental formulations. Corticosteroids (both intranasal and oral) have been associated with significant reductions in eosinophil numbers as well as with reductions in ECP and IL-5 levels in nasal polyps [17,18]. Given the widespread mechanisms of action, it remains still unclear if the clinical benefits of corticosteroids observed in CRSwNP are predominantly mediated solely through the reduction in eosinophil numbers or instead through a combination of this and other anti-inflammatory effects.

Histopathological evidence of inflammation, epithelial fibrosis and basal membrane thickening (BMT) has been shown to be associated with CRS [19,20]. The inflammatory process and its effects could differ between chronic rhino sinusitis with polyps and without polyps. CRSwNPs is characterized by a predominant type 2 inflammatory process mediated by T helper 2 cells and the release of other inflammatory cells such as IL-5, ECP, and eotaxin. It is also characterized by high local levels of IgE [5]. These particular changes lead to increased tissue edema and are responsible for extracellular matrix changes [6]. Basal membrane thickening is correlated with the density of the underlying eosinophils [7]. T helper 2 (TH2) inflammation with eosinophilia, local increase in IgE and tissue edema remodeling are characteristic in CRSwNP (CRS with nasal polyps) patients [21].

Periostin secretion is stimulated by IL-4 and IL-13 secreted from airway epithelial cells, leading to the infiltration of eosinophils [13]. Periostin was reported to have an important role in wound repair and the epithelial–mesenchymal transition of cancer cells. Periostin is present in the thickened basement membrane and in the serum of asthmatic patients, especially those with eosinophilic airway inflammation and atopy, and also induces the hypersecretion of type 2 inflammatory factors, mucus production associated with asthma, allergic rhinitis and CRS [7]. Periostin induces the myofibroblast differentiation of nasal fibroblasts, and this process, along with ECM accumulation, plays a role in the formation of nasal polyps [7]. In normal conditions, periostin is attributed to the wound healing process, but prolonged overexpression of periostin may lead to inflammation of the airways and tissue proliferation [22]. Glucocorticosteroids have been shown to suppress periostin-induced ECM production, and reduce the number of eosinophils and probability of the occurrence of polyps [7]. Stronger expression of periostin is associated with remodeling changes and tissue eosinophilia > 10/HPF [19].

Local IgE formation has been described in nasal polyps, and blocking IgE with medications such as omalizumab is a medical treatment variant for patients with nasal polyps [23]. Treatment with local or systemic glucocorticoids has proven anti-inflammatory effects. They reduce the release of eosinophils by the bone marrow and inhibit their survival time. They have strong vasoconstrictive properties, which reduces swelling and exudate. There are reports suggesting that glucocorticosteroids inhibit periostin expression [7]. We showed that in group 2, treated with prednisone, the level of IgE in the plasma (*p* < 0.0031) decreased significantly one month after the procedure. The strong influence of prednisone on the decrease in IgE reduced tissue edema remodeling, which led to better wound healing.

Total serum IgE levels are elevated in the eosinophilic inflammatory endotype in recurrent CRS [8]. Patients with CRSwNP 80–90% have significant eosinophilia [24]. A study conducted including patients suffering from eosinophilic chronic rhinosinusitis revealed that the cut-off value of 70 mucosal eosinophils/HPF led to the most significant difference in the risk of recurrence in patients treated with FESS [24]. Blood eosinophil counts greater than 0.24109/L are associated with eCRS with tissue eosinophil levels greater than 10 eosinophils per high-power field. It has also been shown that the number of eosinophils in the blood exceeding 0.45109/L is related to the need for long-term treatment with general steroids after endoscopic sinus surgery [8]. Blood eosinophilia testing, which increases the incidence of tissue eosinophilia, should also be considered in patients. These patients would require close observation and intensive conservative treatment to help reduce the risk of reoperation.

We showed that the number of eosinophils in the tissues collected during FESS decreased after treatment with prednisone compared to the first control group (Figure 5, *p* < 0.031) and was responsible for better control of the disease after surgery.

Eotaxin strongly induces the migration or transmigration of eosinophils. High levels of eotaxin in nasal tissues, its strong expression in nasal polyps and stimulation of the expression of endothelial cells result in high levels of eotaxin in patients with eCRS [24]. Eotaxin, interacting with interleukin-5, increases the number of mature eosinophils in the blood, which contributes to the formation of peripheral and tissue eosinophilia. It plays an indirect role in airway remodeling through the recruitment of eosinophils and mast cells, which have profibrogenic and proangiogenic activities, into the site of inflammation [9]. In our research, eotaxin decreased after one week’s treatment with prednisone and one month after surgery (Figure 4, A vs. B *p* < 0.0006, B vs. C *p* < 0.0134). We also observed a lower level of eotaxin in the stroma compared to the control group (*p* limit value < 0.075). Both stroma and plasma eotaxin reduction after prednisone treatment have a potential effect on extracellular matrix remodeling in chronic rhinosinusitis, as well as the reepitelialization process after surgery.

Trials including omalizumab and mepolizumab for treatment in patients requiring reoperation have been conducted, but they were only slightly effective [25].

Prednisone is a low-cost and easily accessible treatment. It promotes patient compliance as once daily dosing is permitted. The use of systemic and local steroids influenced stroma and plasma eotaxin reduction, meaning that the treatment has a potential effect on extracellular matrix remodeling in chronic rhinosinusitis, as well as the reepitelialization process after surgery. We aimed to evaluate the effect of prednisone on nasal and systemic marker expression, as this could provide additional information on the future treatment of chronic rhinosinusitis.

## Figures and Tables

**Table 1:** Median values of inflammation markers and endoscopic scale in groups I and II.

	Group I (Without Oral Steroids, Median Values)			Group II (Treated with Prednisone, Median Values)		
Markers of Inflammation and Endoscopic Scale	One Week before Operation	After Admission to the Hospital	1 Month after Operation	One Week before Operation	After Admission to the Hospital (One Week after Prednisone Treatment)	1 Month after Operation
Periostin in plasma (ng/mL)	26.00	27.68	23.70	32.73	35.67	28.70
Eotaxin in plasma [pg/mL]	131.38	140.64	131.75	163.87	142.09	122.04
IgE in plasma [IU/mL]	55.08	51.16	50.97	47.02	49.94	33.44
Staining score of periostin in epithelium [IRS 0–12]	1.33			0.28		
Staining score of eotaxin in stroma [IRS 0–12]	10			8.43		
Staining score of eosinophils number in tissues	2.47 cells/high-power field			1.64 cells/high-power field		
Lund–Kennedy endoscopic scale	4.13	3.27	1.27	5.14	4.36	0.71

## Data Availability

Not applicable.

## References

[1] Fokkens W.J., Lund V.J., Hopkins C., Hellings P.W., Kern R., Reitsma S., Toppila-Salmi S., Bernal-Sprekelsen M., Mullol J., Alobid I. (2020). European Position Paper on Rhinosinusitis and Nasal Polyps 2020. Rhinology.

[2] Van Crombruggen K., Zhang N., Gevaert P., Tomassen P., Bachert C. (2011). Pathogenesis of chronic rhinosinusitis: Inflammation. J. Allergy Clin. Immunol..

[3] McHugh T., Snidvongs K., Xie M., Banglawala S., Sommer D. (2018). High tissue eosinophilia as a marker to predict recurrence for eosinophilic chronic rhinosinusitis: A systematic review and meta-analysis. Int. Forum. Allergy Rhinol..

[4] Kuhar H.N., Tajudeen B.A., Mahdavinia M., Gattuso P., Ghai R., Batra P.S. (2017). Inflammatory infiltrate and mucosal remodeling in chronic rhinosinusitis with and without polyps: Structured histopathologic analysis. Int. Forum. Allergy Rhinol..

[5] Van Bruaene N., Bachert C. (2011). Tissue remodeling in chronic rhinosinusitis. Curr. Opin. Allergy Clin. Immunol..

[6] Van Bruaene N., Pérez-Novo C.A., Basinski T.M., Van Zele T., Holtappels G., De Ruyck N., Schmidt-Weber C., Akdis C., Van Cauwenberge P., Bachert C. (2008). T-cell regulation in chronic paranasal sinus disease. J. Allergy Clin. Immunol..

[7] Yang H.W., Park J.H., Shin J.M., Lee H.M. (2018). Glucocorticoids ameliorate periostin-induced tissue remodeling in chronic rhinosinusitis with nasal polyps. Clin. Exp. Allergy.

[8] Ho J., Earls P., Harvey R.J. (2020). Systemic biomarkers of eosinophilic chronic rhinosinusitis. Curr. Opin. Allergy Clin. Immunol..

[9] Radajewski K., Wierzchowska M., Grzanka D., Antosik P., Zdrenka M., Burduk P. (2019). Tissue remodelling in chronic rhinosinusitis—Review of literature. Otolaryngol. Pol..

[10] Yamada T., Miyabe Y., Ueki S., Fujieda S., Tokunaga T., Sakashita M., Kato Y., Ninomiya T., Kawasaki Y., Suzuki S. (2019). Eotaxin-3 as a plasma biomarker for & mucosal eosinophil infiltration in chronic rhinosinusitis. Front. Immunol..

[11] Ho J., Hamizan A.W., Alvarado R., Rimmer J., Sewell W.A., Harvey R. (2018). Systemic predictors of eosinophilic chronic rhinosinusitis. Am. J. Rhinol. Allergy.

[12] Ho J., Li W., Grayson J.W., Alvarado R., Rimmer J., Sewell W.A., Harvey R.J. (2021). Systemic medication requirement in post-surgical patients with eosinophilic chronic rhino sinusitis (eCRS). Rhinology.

[13] Remmele W., Stegner H.E. (1987). Recommendation for uniform definition of an immunoreactive score (IRS) for immunohistochemical estrogen receptor detection (ER-ICA) in breast cancer tissue. Pathologe.

[14] Chang M.T., Noel J., Noel F.A., Ayoub N.F., Qian Z.J., Dholakia S., Nayak J.V., Patel Z.M., Hwang P.H. (2021). Oral Corticosteroids Following Endoscopic Sinus Surgery for Chronic Rhinosinusitis Without Nasal Polyposis: A Randomized Clinical Trial. JAMA Otolaryngol. Head Neck. Surg..

[15] Head K., Chong L.Y., Hopkins C., Philpott C., Burton M.J., Schilder A.G. (2016). Short-course oral steroids alone for chronic rhinosinusitis. Cochrane Database Syst. Rev..

[16] Kalish L., Snidvongs K., Sivasubramaniam R., Cope D., Harvey R.J. (2012). Topical steroids for nasal polyps. Cochrane Database Syst. Rev..

[17] Hamilos D.L., Thawley S.E., Kramper M.A., Kamil A., Hamid Q.A. (1999). Effect of intranasal fluticasone on cellular infiltration, endothelial adhesion molecule expression, and proinflammatory cytokine mRNA in nasal polyp disease. J. Allergy Clin. Immunol..

[18] Zhang Y., Lou H., Wang Y., Li Y., Zhang L., Wang C. (2019). Comparison of corticosteroids by 3 approaches to the treatment of chronic rhinosinusitis with nasal polyps. Allergy Asthma Immunol. Res..

[19] Ebenezer J.A., Christensen J.M., Oliver B.G., Oliver R.A., Tjin G., Ho J., Habib A.R., Rimmer J., Sacks R., Harvey R.J. (2017). Periostin as a marker of mucosal remodelling in chronic rhinosinusitis. Rhinology.

[20] Snidvongs K., Lam M., Sacks R., Earls P., Kalish L., Phillips P.S., Pratt E., Harvey R.J. (2012). Structured histopathology profiling of chronic rhinosinusitis in routine practice. Int. Forum. Allergy Rhinol..

[21] Snidvongs K., Chin D., Sacks R., Earls P., Harvey R.J. (2013). Eosinophilic rhinosinusitis is not a disease of ostiomeatal occlusion. Laryngoscope.

[22] Ohta N., Ishida A., Kurakami K., Suzuki Y., Kakehata S., Ono J., Ikeda H., Okubo K., Izuhara K. (2014). Expressions and roles of periostin in otolaryngological diseases. Allergol. Int..

[23] Gevaert P., Calus L., Van Zele T., Blomme K., De Ruyck N., Bauters W., Hellings P., Brusselle G., De Bacquer D., van Cauwenberge P. (2013). Omalizumab is effective in allergic and nonallergic patients with nasal polyps and asthma. Allergy Clin. Immunol..

[24] Tokunaga T., Sakashita M., Haruna T., Asaka D., Takeno S., Ikeda H., Nakayama T., Seki N., Ito S., Murata J. (2015). Novel scoring system and algorithm for classifying chronic rhinosinusitis: The JESREC Study. Allergy.

[25] De Schryver E., Derycke L., Calus L., Holtappels G., Hellings P.W., Van Zele T., Bachert C., Gevaert P. (2017). The effect of systemic treatments on periostin expression reflects their interference with the eosinophilic inflammation in chronic rhinosinusitis with nasal polyps. Rhinology.

